# IL-17 signaling pathway in SPP1^+^ macrophages drives digestive tract cancer progression

**DOI:** 10.1016/j.gendis.2024.101489

**Published:** 2024-12-14

**Authors:** Yihong Wei, Ying Xu, Yanqiong Zeng, Amin Zhang, Xiangling Xing, Wancheng Liu

**Affiliations:** a Department of Hematology, Qilu Hospital of Shandong University, Jinan, Shandong 250012, China; b Eye Hospital of Shandong First Medical University, Jinan, Shandong 250012, China; c National Key Laboratory of Immunity and Inflammation, Suzhou Institute of Systems Medicine, Chinese Academy of Medical Sciences & Peking Union Medical College, Suzhou, Jiangsu 215123, China; d Department of Radiation Oncology, Qilu Hospital of Shandong University, Jinan, Shandong 250012, China

Digestive tract cancers (DTCs) are cancers that occur in the gastrointestinal tract and related organs, including esophageal, gastric, and colorectal cancer. Since these types of cancer share similar endoderm developmental origins, the genomic and other molecular features can possess many similarities. Therefore, it is urgent to explore the commonalities of molecular characteristics and signaling pathways in the tumor microenvironment among these diseases.

To uncover the shared molecular characteristics in DTCs, we obtained the recent RNA sequence profiles from The Cancer Genome Atlas (TCGA) database, encompassing esophageal carcinoma (ESCA), stomach adenocarcinoma (STAD), and colon adenocarcinoma (COAD). Differentially expressed gene (DEG) analysis revealed that ESCA exhibited 376 up-regulated and 626 down-regulated mRNAs, STAD showed 872 up-regulated and 769 down-regulated mRNAs, and COAD had 1123 up-regulated and 939 down-regulated mRNAs (Fig. 1A; Fig. S1A and Table 1). To investigate the existence of co-expression of DEGs in DTCs, we employed Venn diagrams to analyze the overlapping and distinct DEGs in DTCs (Fig. 1B). Subsequently, we explored the relevant signaling pathways associated with these DEGs in the mentioned cancer types using the DAVID tool. Based on the number of genes involved, we prioritized the first eight signal pathways with the highest gene enrichment (Fig. S1B). Additionally, we constructed a protein–protein interaction network complex involving common DEGs to identify key differentially expressed core genes within these cancer types. As shown in Figure 1C, sixteen core genes were identified, of which seven were up-regulated (including CXCL1/5/6/8), indicating that the CXCL family might play a critical role in DTCs.

**Figure 1:**
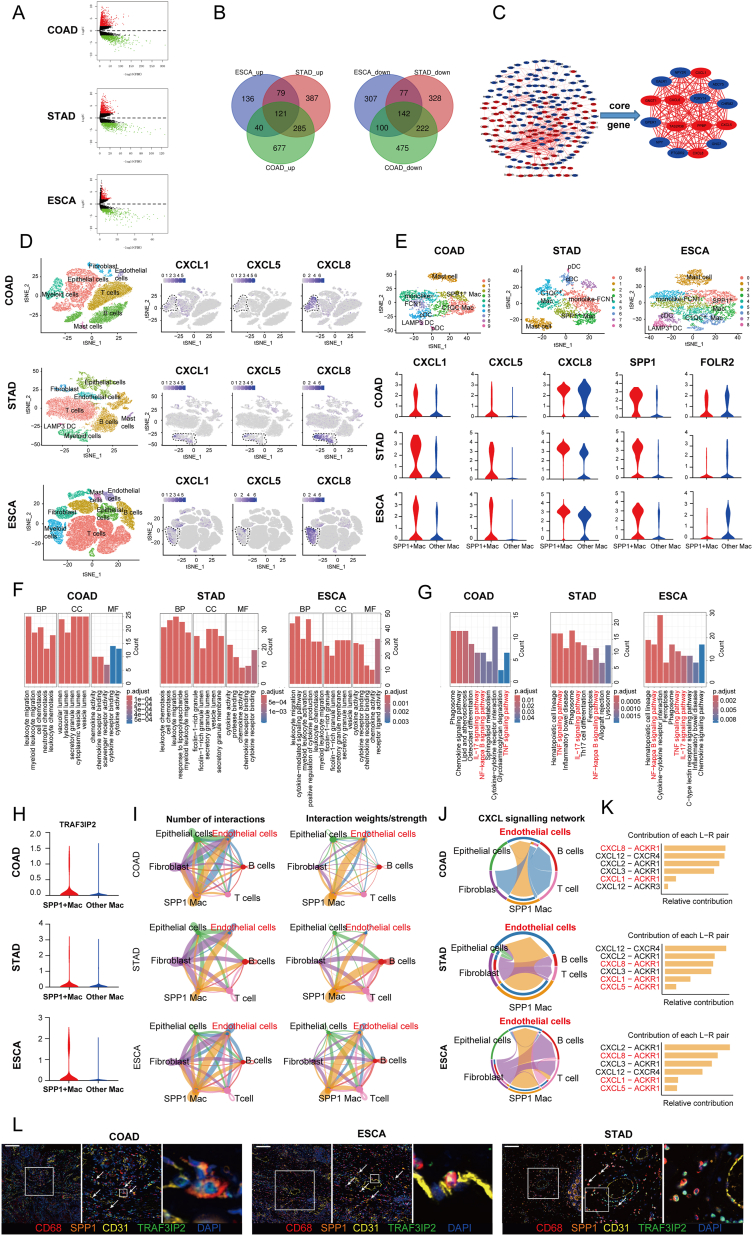
IL-17 signaling pathway in SPP1^+^ macrophages drives digestive tract cancer (DTC) progression. **(A)** The volcano plot of differentially expressed genes (DEGs) in DTCs. **(B)** Co-expression of DEGs in DTCs was detected by Venn diagram. **(C)** Protein–protein interaction analysis of common DEGs by STRING online database and Cytoscape. **(D)** T-distributed stochastic neighbor embedding (t-SNE) plots of DTCs, which are grouped into seven main parts including endothelial cells, fibroblasts, endothelial cells, T/natural killer cells, B cells, mast cells, and myeloid cells. The feature plot of CXCL1, CXCL5, and CXCL8 expression in the seven main groups was shown on the right. **(E)** t-SNE plots of the myeloid cells in DTCs. The violin plot of CXCL1, CXCL5, CXCL8, SPP1, and FOLR2 expression in myeloid cells of DTCs was shown. **(F)** The bar plots showing differences in GO function pathways based on the DEGs of SPP1^+^ macrophages among colon adenocarcinoma (COAD), stomach adenocarcinoma (STAD), and esophageal carcinoma (ESCA). **(G)** The bar plots showing differences in KEGG pathways based on the DEGs of SPP1^+^ macrophages among COAD, STAD, and ESCA. **(H)** Violin plot of TRAF3IP2 expression in myeloid cells of DTCs. **(I)** Interaction net count plot between SPP1^+^ macrophages and other cells. **(J)** The chord plot showing the inferred intercellular communication network of CXCL in COAD, STAD, and ESCA. **(K)** The relative contribution of CXCL receptor ligands in COAD, STAD, and ESCA. **(L)** Representative images of multiplex-immunostaining showed the relationship between SPP1^+^/TRAF3IP2^+^ macrophages and vascular endothelium cells. Scale bar = 100 μm.

The CXCL family is a key group of chemokines capable of inducing leukocyte migration and infiltration. Furthermore, *CXCL1/5/8* have the capacity to stimulate angiogenesis and promote the proliferation of endothelial cells, processes that are intimately linked to tumor initiation and development. To verify the reliability of our bioinformatics analysis results, we collected specimens from cancer patients for verification. The PCR results showed that compared with the paracancerous tissue, the expression of *CXCL1/5/8* was up-regulated in DCT cancer tissue compared with adjacent tissues (Fig. S2). To gain deeper insights into the common features of *CXCL1/5/8* in these three types of cancer, we downloaded scRNA sequencing profiles from the GEO database and conducted an extensive analysis.^1^, ^2^, ^3^ After strict quality control and filtration, we collected 37,768 (COAD), 21,983 (STAD), and 79,261 (ESCA) single cells originating from DTC patients. We classified all cells into 6–10 major clusters representing major cell types, including CD45-negative cells (comprising epithelial cells, fibroblasts, and endothelial cells) and CD45-positive cells (involving T/natural killer cells, B/plasma cells, myeloid cells, and mast cells) (Fig. 1D; Fig. S1C). To gain a deeper understanding of the role of *CXCL1/5/*8 in DTCs, we analyzed the origin of these core genes. Our observation revealed that these genes in DTCs were predominantly found in myeloid cells (Fig. 1D). Consequently, we conducted further sub-clustering of the myeloid cell subtype, employing conventional marker genes to identify dendritic cells, monocytes, macrophages, and mast cells (Fig. S1D). Interestingly, we discovered a significant overlap in the expression of *CXCL1/5/8* with secreted phosphoprotein 1-positive (*SPP1*^+^) macrophages. Additionally, the expression of *CXCL* molecules was mutually exclusive with complement C1q C chain-positive (*C1QC*^+^)/folate receptor beta-positive (*FOLR2*^+^) macrophages (Fig. 1E; Fig. S1E). Although *CXCL1* and *CXCL8* are also expressed in the mono-like FCN1 cell subset, our study was aimed at macrophages. Considering that both *C1QC*^+^/*FOLR2*^+^ and *SPP1*^+^ macrophages may arise from the state of monocyte-like FCN1 cells in the tumor, we believe that this does not affect the fact that SPP1^+^ macrophages are the main source of *CXCL* molecules. Collectively, these findings suggest that *SPP1*^+^ macrophages may serve as the primary source of chemokines *CXCL1*/*5*/*8* within the tumor microenvironment of DTCs. This role is distinct from other macrophages, including *C1QC*^+^/*FOLR2*^+^ macrophages.

With the advancement of scRNA sequencing technology, there is a preference for defining macrophages using *SPP1*, *FOLR2*, and *TREM2*, rather than the traditional M1 and M2.^4^ Considering the critical role of SPP1^+^ macrophages in the DTC tumor microenvironment, we employed the GEPIA2 website to analyze the correlation between the presence of *SPP1*^+^ macrophages and the survival of DTC patients. We selected the top 10 most significant genes to serve as representatives of SPP1^+^ macrophages and assessed their impact on patient survival. The results indicated that higher infiltration of *SPP1*^+^ macrophages led to shorter overall survival in DTC patients (Fig. S1F). Additionally, we conducted Gene Ontology (GO) and Kyoto Encyclopedia of Genes and Genomes (KEGG) enrichment analyses on these three kinds of cancer. The KEGG enrichment analysis revealed the shared pathways in *SPP1*^+^ macrophages among COAD, STAD, and ESCA, including interleukin (IL)-17, nuclear factor-kappa B (NF-κB), and tumor necrosis factor-alpha (TNF-α) signaling pathways. Furthermore, it was observed that the *C1QC*^+^/*FOLR2*^+^ macrophages in DTCs did not exhibit significant enrichment in these pathways, particularly the IL-17 signaling pathway (Fig. 1F, G; Fig. S1G), which indicated that the IL-17 signaling pathway might hold a distinctive role in *SPP1*^+^ macrophages.

It is widely accepted that the IL-17 activity leads to the creation of a pro-tumor microenvironment, relying on the production of inflammatory mediators that reshape the tumor microenvironment by *CXCL1/5/8*. To investigate this further, we evaluated the primary IL-17 receptor (*IL1*7RA) (Fig. S1H). However, it was disappointing to find that there was no significant difference in *IL1*7RA expression between *SPP1*^+^ and *C1QC*^+^/*FOLR2*^+^ macrophages, suggesting that there might be other mechanisms contributing to the differential secretion of *CXCL1/5/8* between these two types of macrophages in the IL-17 signaling pathway. We further estimated other molecules related to IL-17 signaling pathways. TRAF3 interacting protein 2 (*TRAF3IP2*) is the key adaptor molecule directly recruited to IL-17R and is required for both the transcriptional and post-transcriptional changes induced by IL-17.^5^ We found that *TRAF3IP2* was highly expressed in the *SPP1*^+^ macrophages compared with other macrophages (Fig. 1H), suggesting that TRAF3IP2 deficiency might be the reason for the low expression of *CXCL1/5/8* in *C1QC*^+^/*FOLR2*^+^ macrophages. It is the high expression of *TRAF3IP2* that enables *SPP1*^+^ macrophages to play a role as a source of *CXCL1/5/8* in the tumor microenvironment.

Within the tumor microenvironment, the intercellular signaling network plays a vital role in regulating tumor initiation and development. Recognizing the importance of *CXCL1/5/8* in reshaping the vascular network and extracellular matrix to create a conducive environment for tumor cell growth, we employed Cellchat to assess how *SPP1*^+^ macrophages impact other cells within the tumor microenvironment of DTCs. Cellchat allowed us to understand intracellular interactions by predicting protein–protein interactions. The interaction net count plot demonstrated that *SPP1*^+^ macrophages exhibited the highest level of interactions with endothelial cells (Fig. 1I; Fig. S1I). Furthermore, the CXCL signaling pathways between *SPP1*^+^ macrophages and endothelial cells were further analyzed and confirmed (Fig. 1J, K; Fig. S3A). These results suggested that *CXCL1/5/8* secreted by *SPP1*^+^ macrophages might play a significant role in communicating with vascular endothelial cells, enhancing angiogenesis, and exerting their tumor-promoting effects in DTCs. Additionally, we utilized multiple fluorescence immunohistochemistry to detect the relationship between *SPP1*^+^ macrophages and endothelial cells. Our results indicated that there were many *SPP1*^+^ macrophages near the vascular endothelium, which also highly expressed *TRAF3IP2* (Fig. 1L). These results also suggested that *SPP1*^+^ macrophages might play a role in promoting the progression of cancer through communication with vascular endothelial cells.

In summary, our study first establishes the connection between *SPP1*^+^ macrophages and *CXCL1/5/8* in DTCs (Fig. S4). SPP1^+^ macrophages constitute the primary source of *CXCL1*/*5*/*8* in DTCs. Furthermore, we speculated that *TRAF3IP2* might serve as the crucial adaptor protein for the production of *CXCL1/5/8* and exhibits high expression exclusively in *SPP1*^+^ macrophages, which have distinctive functions in the IL-17 signaling pathway. Consequently, *SPP1*^+^ macrophages can recruit myeloid cells and augment angiogenesis through *CXCL1/5/8*, thereby fostering a cancer-promoting microenvironment. Targeting *TRAF3IP2* presents a novel therapeutic strategy for DCTs.

## Ethics declaration

Our study was approved by the Medical Ethical Committee of Qilu Hospital of Shandong University (KYLL-202311-043) and consent was gained from all participants.

## Funding

This work was supported by the National Science Funds of China (No. 82300219), and the 10.13039/100017445Natural Science Foundation of Shandong Province, China (Youth Program; No. ZR2023QH249).

## CRediT authorship contribution statement

**Yihong Wei:** Formal analysis. **Ying Xu:** Methodology. **Yanqiong Zeng:** Writing – original draft. **Amin Zhang:** Data curation. **Xiangling Xing:** Writing – original draft. **Wancheng Liu:** Conceptualization, Funding acquisition.

## Data availability

The single-cell RNA sequencing datasets analyzed during the current study are available in the GEO repository (GSE166555 for COAD, GSE167297 for STAD, and GSE160269 for ESCA). For other original data, please contact the corresponding author.

## Conflict of interests

The authors declared no competing interests.
