# Supplementary material for: IL-17 signaling pathway in SPP1+ macrophages drives digestive tract cancer progression
Source: Genes Dis. 2024 Dec 14;12(4):101489. doi: 10.1016/j.gendis.2024.101489 (PMC11981743; doi:10.1016/j.gendis.2024.101489)
Supplement: Multimedia component 1 [file mmc1.doc]

**Materials and Methods:**

**Single-cell transcriptome analysis**

The scRNA-seq datasets analyzed during the current study are available in the GEO repository, number GSE200997(COAD), GSE167297(STAD) and GSE160269(ESCA). We downloaded matrix file from the GEO dataset for subsequent bioinformatics analysis. We utilized Seurat (version 4.0.2, R version 4.3.2) for downstream analysis. The exclusion criterion were cells more than 3000, and/or expressed genes fewer than 500, and/or mitochondrial percentage >5% in the database. After normalizing the expression matrix, we calculated highly variable genes in cells using the Seurat FindVariableGenes method. Principal component analysis was performed using the highly variable genes, and the top 20 significant principal components were selected for tSNE dimension reduction. To identify the DEGs (Differentially Expressed Genes) in cells, we used FindAllMarkers (Seurat) and conducted the analysis using the Wilcox test.

**Cell type determination and functional enrichment**

For cell annotation, we used the CellMarker website and canonical marker genes as reference objects to determine cell types. Gene Ontology (GO) and Kyoto Encyclopedia of Genes and Genomes (KEGG) analyses were conducted using clusterProfiler based on the DEGs for *SPP*1^+^ macrophages and other macrophages.

**Cell Communication Analysis**

For the analysis of cell-cell communication, CellChat (an R package contained ligand-receptor interaction databases for human and mouse) was utilized to assess the primary signaling inputs and outputs between *SPP*1^+^ macrophages and other cells. After projecting these interactions onto the protein-protein interaction (PPI) network, we used the netVisual_circle (CellChat) function to visualize the strength of cell-cell communication networks between *SPP*1^+^ macrophages and other cells. Additionally, the netVisual_bubble function was used to visualize bubble plots of ligand-receptor interactions between *SPP*1^+^ macrophages and other cells.

**Quantitative real-time PCR**

The cancer and paracanerous tissue of three types cancer were collected from Qilu hospital. The patient's tissue was removed and cleaned during the operation and stored in -80 freezer until further use. The mRNA were extracted using TRIzol followed by reverse transcription into cDNA with the Prime Script RT reagent Kit Perfect Real Time (Takara Bio, Japan). Subsequently, RT-qPCR was conducted using the LightCycler 480II real-time PCR system manufactured by Roche (Switzerland). Primers (BioSune Company, China) were shown as follows: GAPDH (Homo): forward: GCT CTC TGC TCC TCC TGT TC, reverse: GTT GAC TCC GAC CTT CAC CT. *CXCL*1 (Homo): forward: TTG CCT CAA TCC TFC ATC CC, reverse: GTT GGA TTT GTC ACT GTT CAG CAT. *CXCL*5 (Homo): forward: GAC CAC GCA AGG ACT TCA TC, reverse: GGA GGC TAC CAC TTC CAC CT. *CXCL*8 (Homo): forward:CTC TGT GTG AAG GTG CAG TTT T, reverse: GTT TTC CTT GGG GTC CAG ACA.

**Multichannel imaging and Image analysis**

Multichannel imaging was conducted using the Vectra Polaris Imaging System (Akoya Biosciences). Slides were meticulously captured at a magnification of ×200, and subsequent image analysis was carried out utilizing QuPath V.0.4.3. The segmentation of cells employed an algorithm predicated on the presence of nuclear DAPI staining. Subsequently, the fluorescence staining intensity of cells was quantified for each specific marker. The primary antibodies were shown as follow: anti-CD31 (Abcam, AB182981), anti-CD68 (Abcam, AB213363), anti-*TRAF3IP2* (Affinity Biosciences, DF14487), *SPP*1(Proteintech, 25715-1-AP).

**Figure Legend:**

**Supplementary Figure1: Landscape of scRNA transcriptome DCTs. (A):** The heatmap of DEGs (mRNA) in DTCs. **(B):** Bar plots showing differences in GO function pathways based on the DEGs among DTCs. **(C):** Dot plot showing the conventional marker genes in each sub-cluster. The color and size indicate the effect size in cells of DTCs. **(D):** Dot plot showing the significant marker genes in each sub-cluster of myeloid cells in DTCs. The color and size indicate the effect size. **(E):** Feature plot of *CXCL*1, *CXCL*5, *CXCL*8, *SPP*1 and *FOLR2* expression of myeloid cells in DTCs. **(F):** Kaplan-Meier curve showing DTCs patients survival with different *SPP*1^+^ macrophages infiltration. **(G):** Bar plots showing differences in GO function pathways based on the DEGs of *C1QC*^+^/*FOLR2*^+^ macrophage among COAD, STAD and ESCA. **(H):** Violin plot of IL17RA expression between *SPP*1^+^ macrophage and *C1QC*^+^/*FOLR2*^+^ cells in DTCs. **(I):** Cell-Cell communications between *SPP*1^+^ macrophage and other cells.

**Supplementary Figure2: The mRNA expression of *CXCL*s in DCT patients. (A):** The mRNA expression of *CXCL1/5/8* in 6 DTC patients (two patients per type of cancer, including tumor and normal adjacent tissue).The left panel is two STAD patients, 2 COAD patients in the middle panel, and 2 ESCA patients on the right panel. ***P< 0.001

**Supplementary Figure3: Communication patterns of *SPP*1^+^ macrophages. (A):** Dot plot exhibited incoming communication of *SPP*1^+^ macrophages.

**Supplementary Figure4:** Schematic diagram of the role of *SPP*1^+^ macrophages in the DCTs’ TME.

**TABLE1 | Co-expression of mRNAs in DCTs.**

| Expression | Gene name |
| --- | --- |
| Co-upregulation  of mRNA | ADAM12; PLAU; SLC28A3; MMP13; IGF2BP3; HOXC13; SLCO1B3; COL1A1; ANLN; GFY; ALX1; MUC16; CREG2; SAA2-SAA4; TNFRSF11B; BIRC7; EN1; RIPPLY3; TNNI3; PPBP; STRIP2; PIWIL1; CLDN6; CST1; CEL; MYBL2; *CXCL*8; PAEP; DCSTAMP; SIX3; MCEMP1; KISS1R; DLX4; IL17C; FGF19; DLX6; CCL26; UCN2; C5orf46; *SPP*1; OLR1; SLCO1B1; CILP2; MAGEA3; ACAN; DMBX1; GJB7; SPERT; RFX8; ULBP1; SLC6A14; PCSK9; GNGT1; IBSP; TRIP13; CBX2; ADAMTS12; MMP11; ZNF695; SKA3; NXPH4; PSG9; CST4; ACTL8; MMP3; FAP; INHBA; SMKR1; BLACAT1; HOXC12; UBE2C; MEX3A; ERFE; WNT2; FOXL2; POU6F2; MUCL1; NKD2; GABRD; WDR72; MAGEA6; *CXCL*1; MFAP2; STRA6; MAGEA1; SALL4; MAT1A; SERPINE1; IGFL3; EVA1A; MAGEA11; *CXCL*5; CLEC5A; HOXC11; *CXCL*6; SAGE1; AHSG; SIX1; SP8; CST2; COL10A1; IL11; SHOX2; CSAG2; SAA2; COL11A1; TAS2R38; PRAME; CTHRC1; RECQL4; MMP10; CSAG3; GAD1; GABRA3; C20orf204; ZNF280A; DUSP9; TMEM270; CSF2; XRCC2; ESM1; |
| Co-downregulation  of mRNA | HPSE2; KCNMA1; HSPB6; FGL2; CHRDL1; LDB3; RBFOX3; PLIN4; CNN1; PLN; SYT10; FRRS1L; REP15; PIRT; CADM2; TNXB; ADCY5; NCAM1; MAL; GALR1; CLEC3B; MT1M; ENAM; TAFA4; LONRF2; KCNJ13; FHL1; PHOX2B; RSPO2; SCUBE1; GNAO1; MFSD4A; JPH2; CMA1; LMOD1; ZBTB16; TCEAL6; KCNMB1; DES; ASPA; TACR2; MYH11; NPAS4; C1QTNF7; NEGR1; SULT2A1; NPY; COL19A1; ABI3BP; FAM180B; SCNN1B; CALY; HPGD; RBPMS2; SCGB2A1; PDK4; CNKSR2; GFRA2; ADCYAP1R1; CHAD; PTGDR2; DPP6; ANGPTL1; TMEM100; P2RY14; ASB11; DPT; SORCS1; GUCA2B; SCARA5; TCEAL2; SLC5A7; ATP1A2; ADGRB3; ADHFE1; CADM3; SORBS1; MYLK; PRIMA1; SGCA; GRIK5; LYVE1; PMP2; LGI1; C16orf89; NECAB1; MYOC; VIP; GPER1; PDZRN4; CARTPT; SLC2A4; P2RX2; KCNA5; GRIK3; TREH; VSTM2A; GNG7; CHRM2; GCNT4; CMTM5; SCN7A; ACTG2; TMOD1; LCN6; ADH1B; KCTD8; KCNB1; KCNE2; NPTX1; NPY2R; NEXMIF; PPP1R1A; VEGFD; FAM107A; PGM5; FXYD1; VSIG2; ASB5; GFRA1; SPHKAP; GSTM5; SIGLEC11; MMRN1; PI16; ALDOB; GRIA4; SCNN1G; CASQ2; PYGM; HSPB7; PLP1; PTCHD1; MAMDC2; XKR4; KCNJ16; STMN2; SYNM; C2orf40; CFD; SMYD1; JCHAIN; |
