# Supplementary figures and images for: IL-17 signaling pathway in SPP1+ macrophages drives digestive tract cancer progression
Source: Genes Dis. 2024 Dec 14;12(4):101489. doi: 10.1016/j.gendis.2024.101489 (PMC11981743; doi:10.1016/j.gendis.2024.101489)

A

■ Paracancerous tissue  
■ Cancer tissue

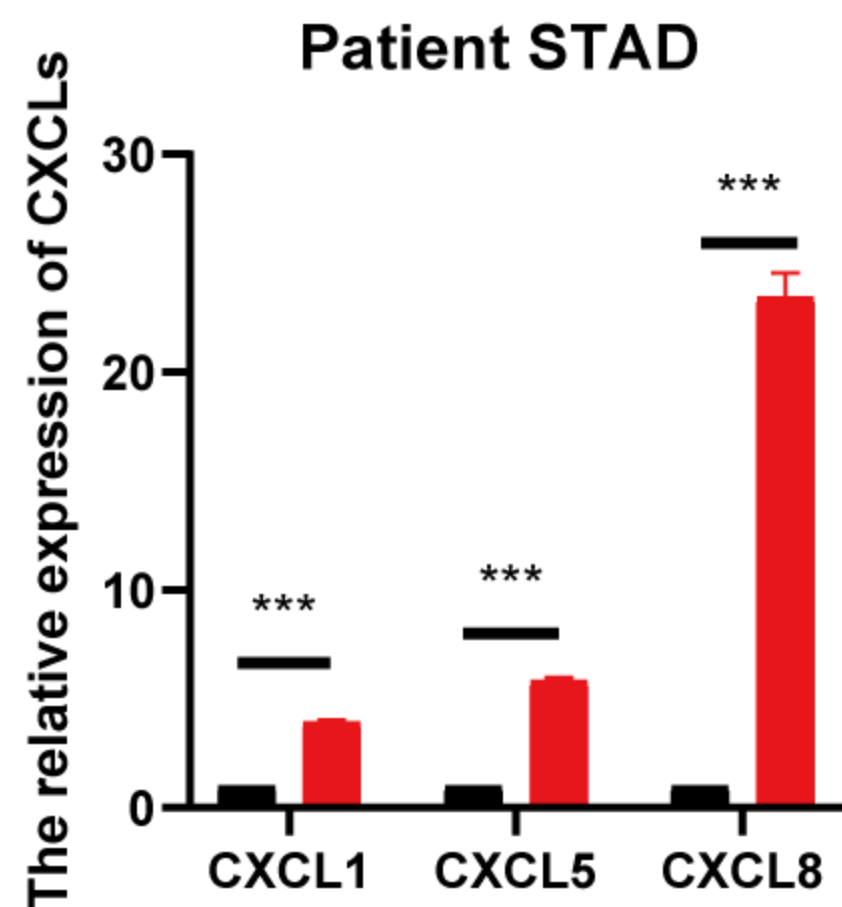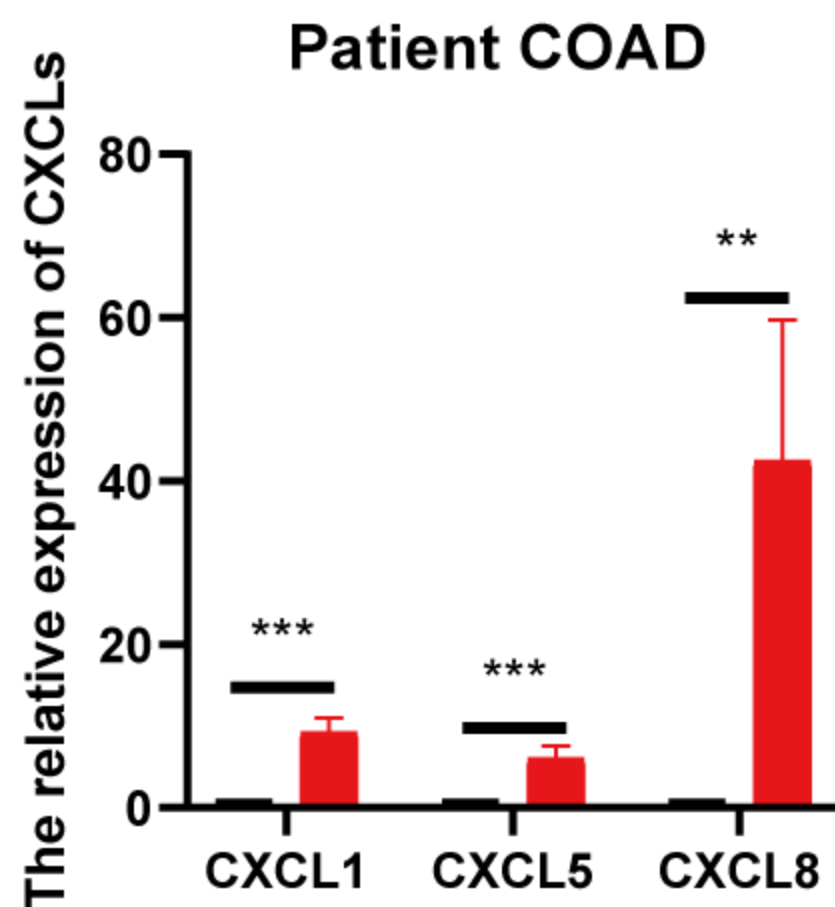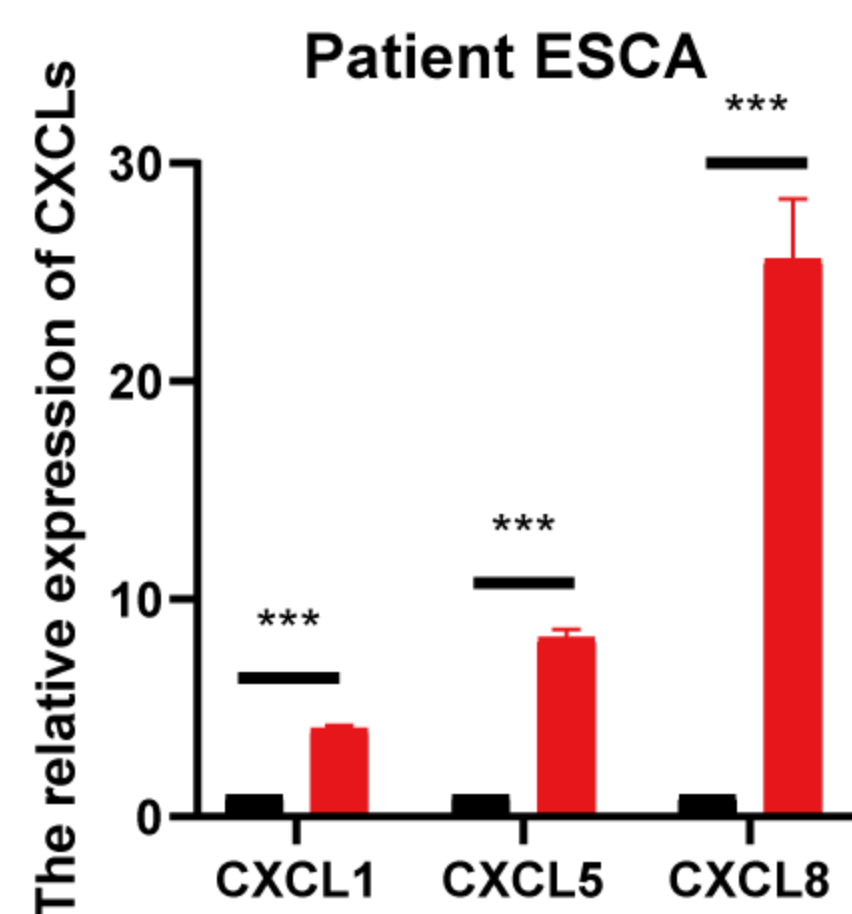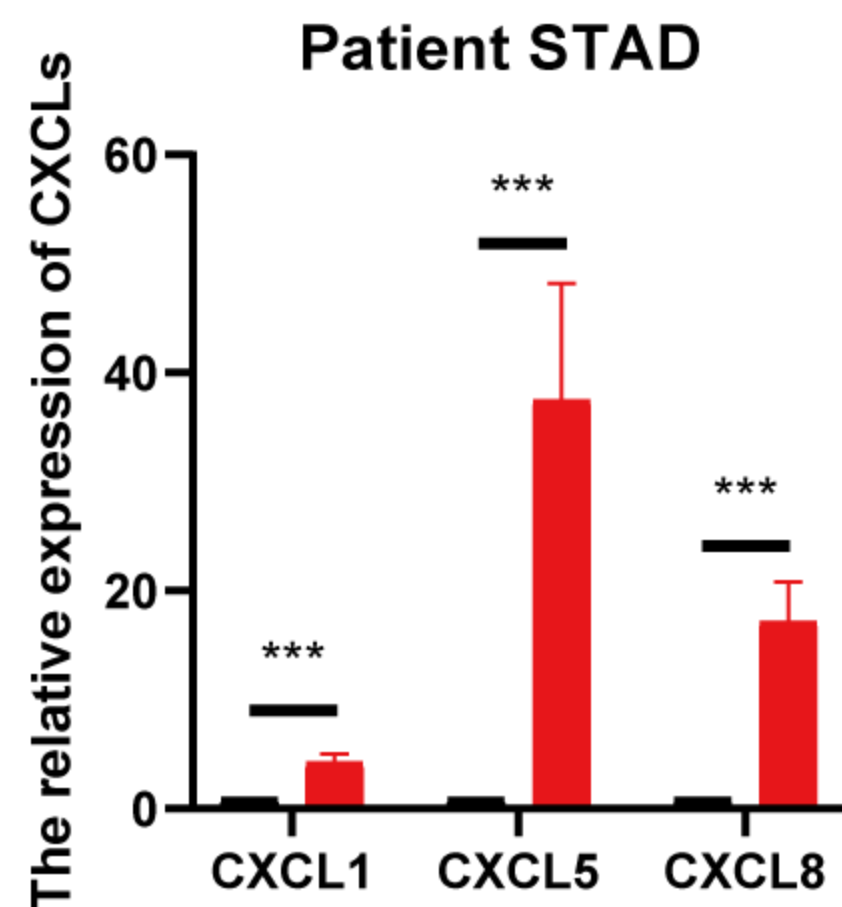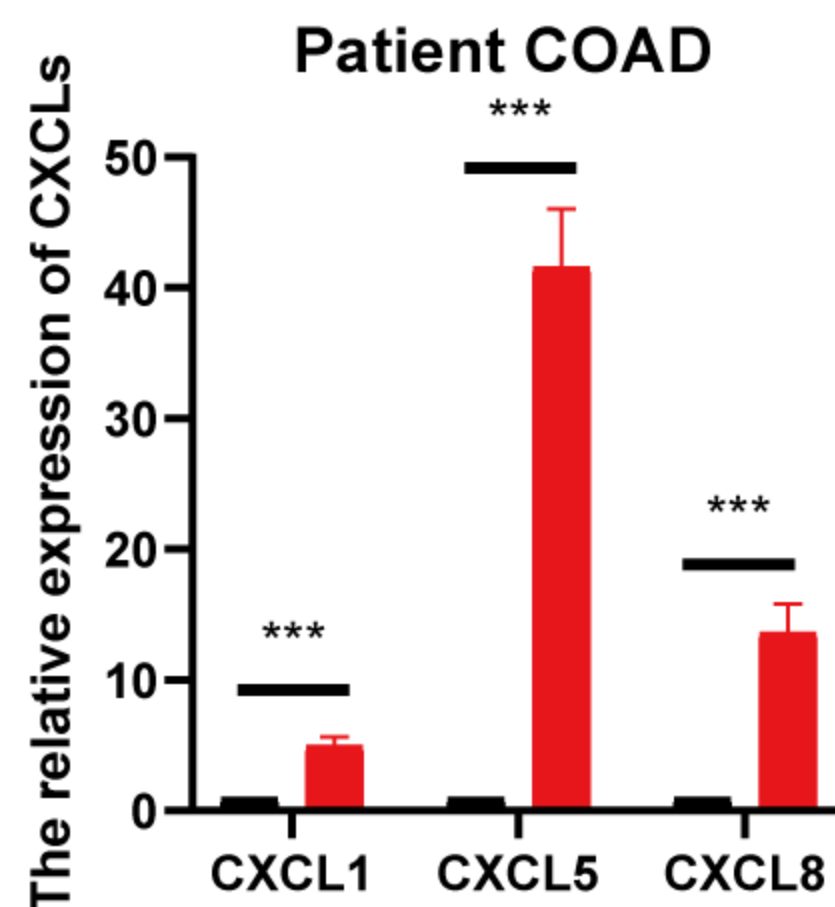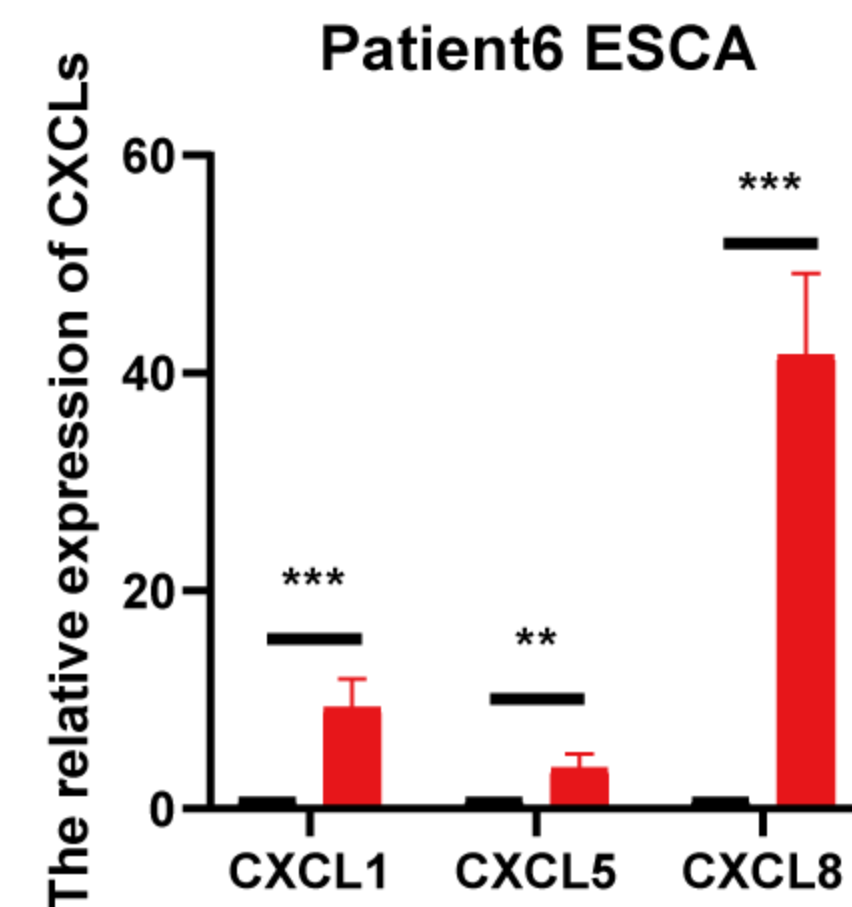

Supplement: Multimedia component 3 [file mmc3.pdf]

A

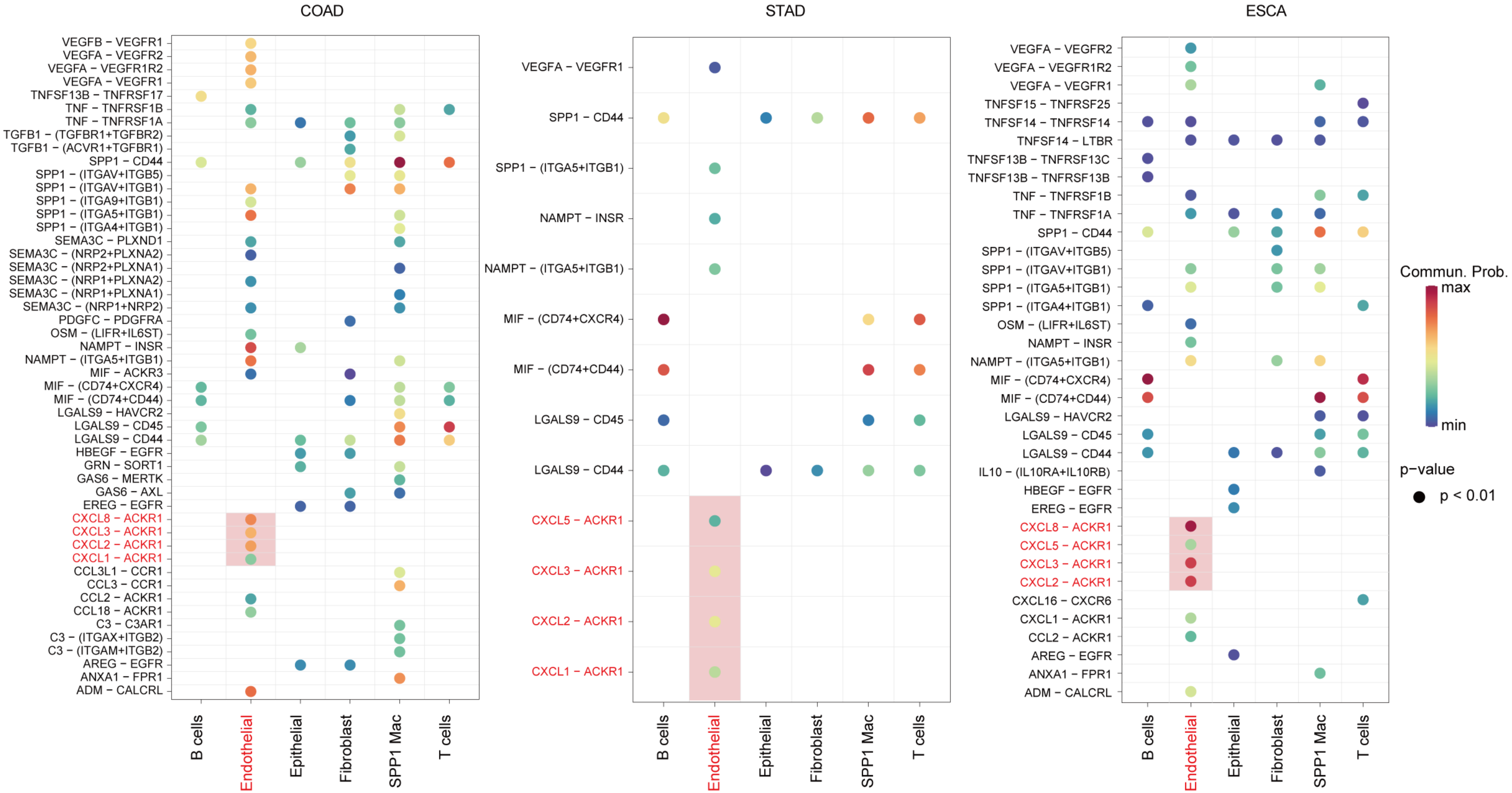

Supplement: Multimedia component 4 [file mmc4.pdf]

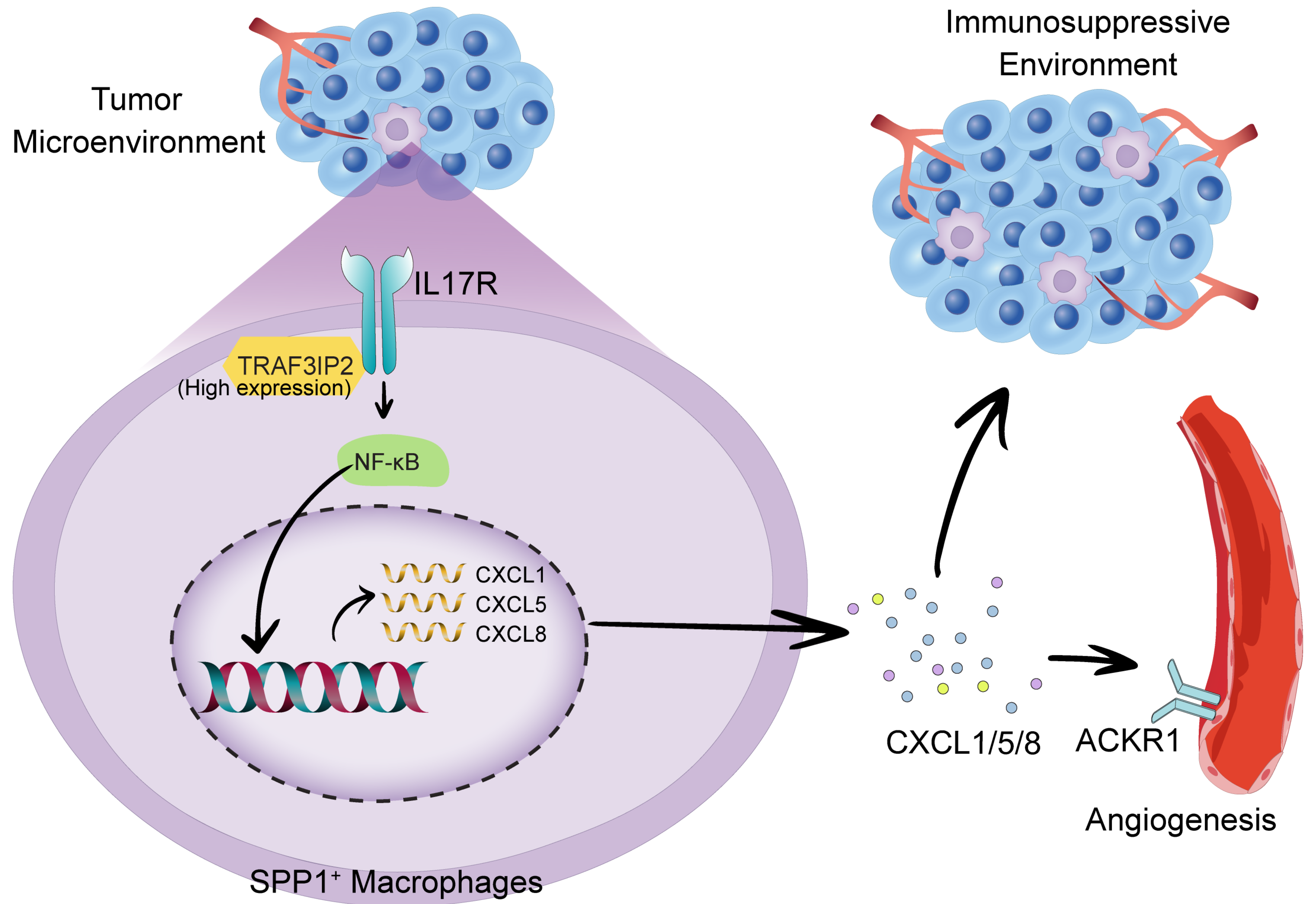

Supplement: Multimedia component 5 [file mmc5.pdf]
