# Supplementary material for: IL-17 signaling pathway in SPP1+ macrophages drives digestive tract cancer progression
Source: Genes Dis. 2024 Dec 14;12(4):101489. doi: 10.1016/j.gendis.2024.101489 (PMC11981743; doi:10.1016/j.gendis.2024.101489)
Supplement: Multimedia component 2 [file mmc2.pdf]

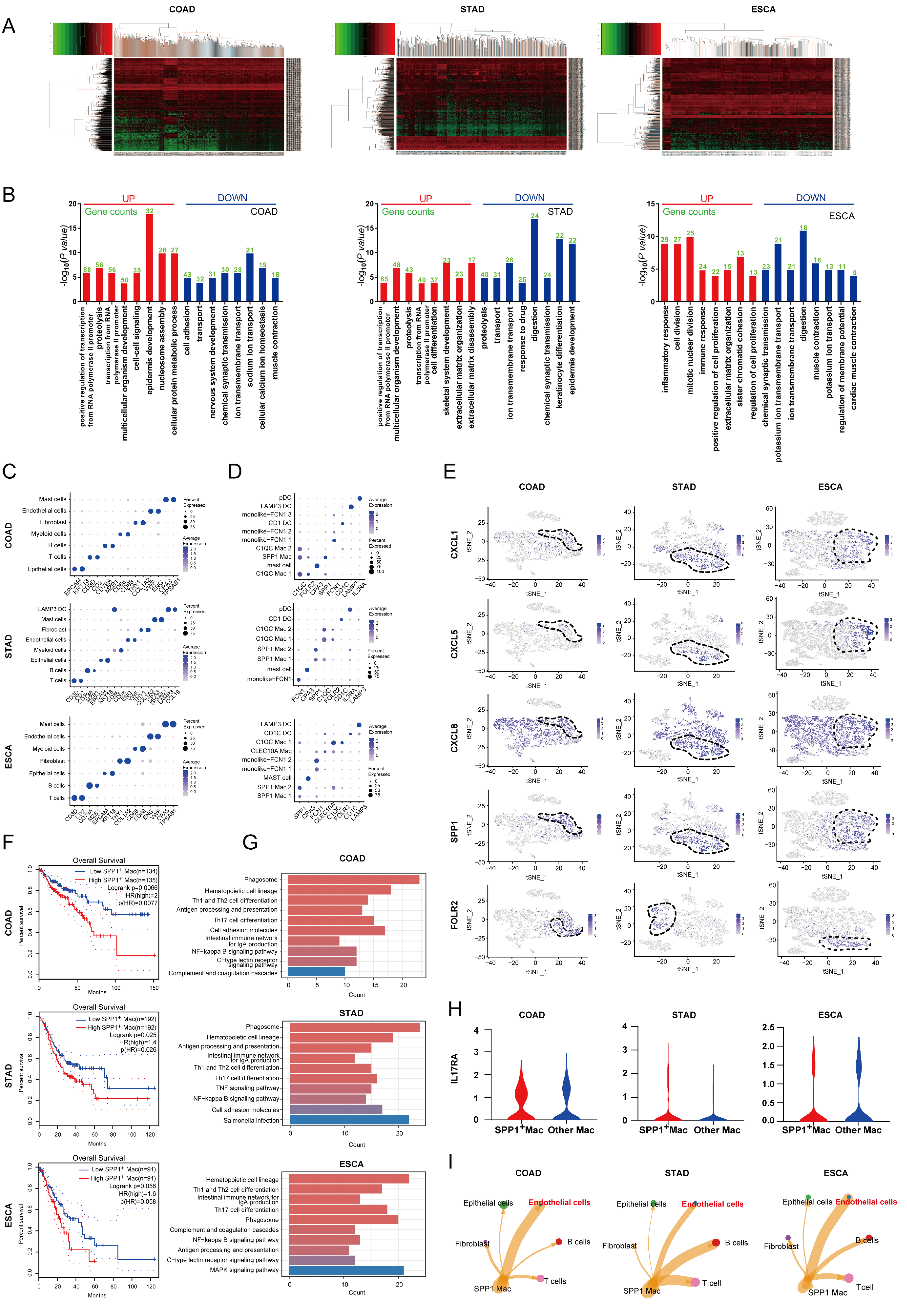

A
COAD
STAD
ESCA
B
UP
DOWN
Gene counts
-log10(P value)
positive regulation of transcription from RNA polymerase II promoter
proteolysis
transcription from RNA polymerase II promoter
multicellular organism development
cell-cell signaling
epidermis development
nucleosome assembly
cellular protein metabolic process
cell adhesion
transport
nervous system development
chemical synaptic transmission
ion transmembrane transport
sodium ion transport
cellular calcium ion homeostasis
muscle contraction
cell differentiation
skeletal system development
extracellular matrix organization
extracellular matrix disassembly
response to drug
digestion
keratinocyte differentiation
inflammatory response
cell division
mitotic nuclear division
immune response
positive regulation of cell proliferation
sister chromatid cohesion
regulation of cell proliferation
potassium ion transmembrane transport
potassium ion transport
regulation of membrane potential
cardiac muscle contraction
C
D
E
CXCL1
CXCL5
CXCL8
SPP1
FOLR2
tSNE_1
tSNE_2
F
Overall Survival
Low SPP1+ Mac(n=134)
High SPP1+ Mac(n=135)
Logrank p=0.0066
HR(high)=2
p(HR)=0.0077
Low SPP1+ Mac(n=192)
High SPP1+ Mac(n=192)
Logrank p=0.025
HR(high)=1.4
p(HR)=0.026
Low SPP1+ Mac(n=91)
High SPP1+ Mac(n=91)
Logrank p=0.056
HR(high)=1.6
p(HR)=0.058
Percent survival
Months
G
Phagosome
Hematopoietic cell lineage
Th1 and Th2 cell differentiation
Antigen processing and presentation
Th17 cell differentiation
Cell adhesion molecules
Intestinal immune network for IgA production
NF-kappa B signaling pathway
C-type lectin receptor signaling pathway
Complement and coagulation cascades
TNF signaling pathway
Salmonella infection
MAPK signaling pathway
Count
H
IL17RA
SPP1+Mac
Other Mac
I
Epithelial cells
Endothelial cells
Fibroblast
B cells
T cells
SPP1 Mac
